# The impact of IgM deposits on the outcome of Nephrotic syndrome in children

**DOI:** 10.1186/s12882-017-0674-7

**Published:** 2017-08-03

**Authors:** Sandra Juozapaite, Rimante Cerkauskiene, Arvydas Laurinavicius, Augustina Jankauskiene

**Affiliations:** 1 Institute of Clinical Medicine, Faculty of medicine, Vilnius University, M.K.Ciurlionio srt. 21, 03101 Vilnius, Lithuania; 2 Department of Pathology, Forensic Medicine and Pharmacology, Faculty of Medicine, Vilnius University, Vilnius, Lithuania; 3 National Center of Pathology, affiliate of Vilnius University Hospital Santariskiu Clinics, P. Baublio str. 5, 08406 Vilnius, Lithuania

**Keywords:** Nephrotic syndrome, IgM deposition, Renal biopsy, Minimal change disease, Focal segmental glomerulosclerosis

## Abstract

**Background:**

The significance of IgM deposits in glomerular mesangium has been controversial since they were first described due to the variations in the both the definitions used and described impact on clinical outcome. The aim of our study was to evaluate the significance of the IgM deposits in the glomerular mesangium for outcomes of nephrotic syndrome (NS) in children.

**Methods:**

Forty-five children with NS who underwent renal biopsy at tertiary pediatric hospital from January 1st, 2000 to December 31st, 2015 and the pathology diagnosis of minimal change disease, focal segmental glomerulosclerosis and mesangial hypercellularity (MH) were retrospectively analyzed. IgM positivity was defined as ≥1+ imunofluorescence with predominantly mesangial distribution. The patients were stratified into IgM-positive (*n* = 18) and IgM-negative (*n* = 27).

**Results:**

At the end of the median follow-up 4.5 years (range 0.17–13.14), the IgM-positive group was represented by 11 patients (61.1%) in remission, 3 patients (16.7%) with active disease and normal kidney function, 2 (11.1%) patients with active disease and impaired kidney function, 2 (11.1%) patients on renal replacement therapy. Accordingly, the IgM-negative group included 13 patients (48.1%) in remission, 12 (44.4%) with active disease and normal kidney function, 1 (3.7%) with active disease and impaired kidney function, 1 (3.7%) on renal replacement therapy, with no statistical significance between groups (*p* = 0.186).

**Conclusions:**

This study did not reveal significant differences of the disease outcomes between IgM-positive and IgM-negative groups.

## Background

The incidence of nephrotic syndrome (NS) is 2–7 cases per 100,000 children per year [1]. The most common histological findings in children presenting with NS are minimal change disease (MCD), focal segmental glomerulosclerosis (FSGS) and membranous nephropathy (MN) [1]. Immunoglobulin M (IgM) nephropathy was first described in 1978 by Cohen [2] and Bhasin [3], who reported 12 and 11 patients respectively, presenting with heavy proteinuria and the predominant IgM deposits in the glomeruli. The rate of IgM nephropathy ranges from 2 to 18.5% because of different diagnostic definitions [4–8]. Since its description, it has been an object for debate because of various definitions and impact on clinical outcome. The significance of IgM deposits is controversial. Some authors suggested that IgM deposits could be passively trapped in the glomeruli [9] while others considered it as a new clinically distinct entity [2, 10–12].

The aim of our study was to evaluate the significance of IgM deposits in the glomerular mesangium for the clinical course, treatment strategy and outcomes of NS in children and to evaluate the histological disease progression based on follow-up biopsies.

## Methods

### Patient selection

**Fig. 1:**
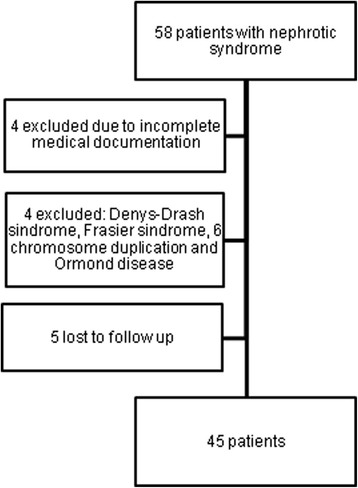
Patient selection chart

A retrospective chart analysis was done on children (age 0–18 years) with NS who underwent renal biopsy at Children‘s Hospital, Affiliate of Vilnius University Hospital Santariskiu Clinics from January, 2000 to December, 2015 with the histological diagnosis of MCD, FSGS and MH. Indications for renal biopsy were: patient’s age < 1 year or >10 years at the time of first manifestation of NS or additional clinical features (hematuria, arterial hypertension, decreased kidney function or extrarenal symptoms), and a frequently relapsing, steroid-dependent or steroid-resistant form of NS. Patients with systemic disease, causing IgM deposition in the kidney, were excluded from this study. Detail of the patient selection process is presented in Fig. 1.

Patients were divided into two groups, IgM positive and IgM negative to compare the patient’s age, gender, clinical manifestation, treatment strategy, response to steroids and outcome. The median follow up time was 4.5 years (range 0.17–13.14).

### Renal biopsies

All biopsies were obtained by the same investigator and evaluated by light and immunofluorescence (IF) microscopy by the same pathologist, with 23 biopsies also evaluated by electron microscopy.

Minimal change NS was defined as edema or nephrotic range proteinuria (≥ 3+ urine dipstick test) and hypoalbuminemia ≤25 g/l, and pathology findings of minimal change disease on light microscopy with or without mesangial proliferation, C1q and/or IgM mesangial deposition on IF microscopy, and with or without foot process effacement on electron microscopy.

FSGS was characterized by at least one segmental lesion, with obliteration of capillaries with or without adhesion to the Bowman’s capsule.

MH was defined as a uniform increase in mesangial cells (more than 3 mesangial cells per mesangial area) in more than 80% of the glomeruli [13].

**Fig. 2:**
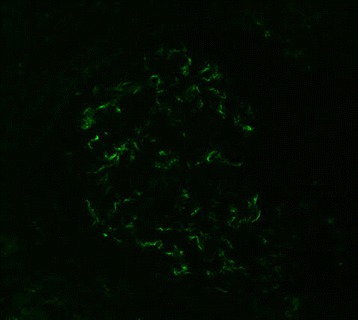
Weak-to-moderate IgM deposition in the mesangium by direct immunofluorescence

Glomerular IF findings were graded on a scale 0–4+. IgM positivity was defined as a grade of ≥1+ IF intensity with predominantly mesangial distribution (Fig. 2).

### Diagnostic definitions

Arterial hypertension was defined as systolic and/or diastolic blood pressure elevation above the 95th percentile based on the child’s age, gender and height or a patient currently on antihypertensive medication [14].

Hematuria was defined as ≥1+ on dipstick test or >5 red blood cells present per high power field of spun urinary sediment.

Glomerular filtration rate (GFR) was calculated using the Schwartz formula [15]. The value of GFR < 90 ml/min/1.73 m^2^ was defined as decreased kidney function. Serum creatinine was measured by Jaffe method during the study period.

Definitions used to evaluate response to therapy based on KDIGO 2012 guidelines [16]:

- Remission – < 1+ of protein on urine dipstick test for 3 consecutive days
- Relapse – occurrence of >3+ protein on urine dipstick for 3 consecutive days for a patient who was previously in remission
- Steroid-dependent nephrotic syndrome – patients who relapsed during the steroid treatment or within 2 weeks after the discontinuation of steroids
- Steroid-resistant nephrotic syndrome – persistent proteinuria for ≥4 weeks while treated with steroids.
- Frequently relapsing nephrotic syndrome – patients who had 2 or more relapses in 6 months or 4 or more relapses in 12 months during the first year of treatment.

Definitions used to evaluate patient outcomes:

- Active disease – persistent proteinuria and/or decreased kidney function during the last visit. This is divided to active disease with normal kidney function or active disease with decreased kidney function.
- Persistent proteinuria – any amount of protein found in urine with dipstick test at the time of last follow-up.
- Kidney replacement therapy – transplantation or dialysis.
- Cured – a patient who was relapse-free for at least 5 years.

### Statistical analysis

Statistical analysis was performed using the SPSS 23.0 software version for Windows (SPSS Inc., Chicago, IL, USA). All data are reported as median (range) unless otherwise specified. Categorical variables are described as frequency and percentage. Means of quantitative variables were compared using the t-test or Mann-Whitney test if data is not normally distributed. Categorical variables were compared using the Person’s chi square test, Fisher exact test or likelihood ratio, as appropriate. A *p* value of <0.05 was considered to be statistically significant.

## Results

### Biopsy results

Forty-five patients were included into the final analysis: 18 patients (40%) were IgM positive and 27 (60%) were IgM negative. Initial kidney biopsies were performed at a median of 0.71 years (0.1–10.03) in the IgM positive group and a median of 1.00 years (0.1–7.6) in the IgM negative group after the disease presentation with no statistically significant difference (*p* = 0.95). Detailed biopsy results are displayed in Table 1.

**Table 1:** Demographic and clinical data at the onset of the disease

	IgM + (*n* = 18)	IgM – (*n* = 27)	*p* value
First manifestation			
Age, years^a^	4.51 (0.96–14.23)	4.09 (1.02–17.05)	0.926
Gender, n (%)			0.371
Boys	12/18 (66.7)	14/27 (51.9)	
Girls	6/18 (33.3)	13/27 (48.1)	
Clinical data, n (%)			
Hematuria	13/18 (72.2)	17/27 (63.0)	0.748
Hypertension	7/18 (38.9)	11/27 (40.8)	1
Decreased GFR	2/18 (11.1)	5/27 (18.5)	0.684
Extrarenal symptoms	5/18 (27.8)	2/27 (7.4)	0.098
Primary response to steroids, n (%)			
Sensitive	10/18 (55.6)	15/27 (55.6)	1
Dependent	2/18 (11.1)	7/27 (25.9)	0.279
Resistant	6/18 (33.3)	5/27 (18.5)	0.304
Treatment, n^b^(%)			
Only steroids	3/18 (16.7)	8/27 (29.6)	0.482
Cumulative dose of steroids	523.3 mg/kg	429.6 mg/kg	0.8
Cyclosporine	15/18 (83.3)	18/27 (66.7)	0.308
Mycophenolate mofetil	4/18 (22.2)	4/27 (14.8)	0.694
Cyclophosphamide	6/18 (33.3)	9/27 (33.3)	1
Rituximab	3/18 (16.7)	1/27 (3.7)	0.286
Levomisole	3/18 (16.7)	4/27 (14.8)	1
Biopsy results, n (%)			0.049
Minimal change disease	8/18 (44.4)	16/27 (59.3)	
Mesangial hypercellularity	1/18 (5.6)	6/27 (22.2)	
Focal segmental glomerulosclerosis	9/18 (50.0)	5/27 (18.5)	

^a^Values are given as median, with the range in parenthesis

^b^Total number of patients treated with each drug

Imunofluorescence findings: out of 18 IgM positive biopsies, 11 were IgM 1+ positive (61.1%), 5 were IgM 2+ positive (27.8%) and 2 were IgM 3+ positive (11.1%). 8 (44.4%) biopsies contained other deposits: 6 had C3 deposits (33.3%), also IgA and IgG deposits were each present in 4 (22.2%) biopsies. Electron microscopy was performed for 23 biopsies. Electron dense deposits were found in 3 biopsies (33.3%), all in IgM positive group

### Disease presentation and clinical course

Demographic data and clinical manifestation did not differ significantly between the groups (Table 1.) Five (27.8%) patients in the IgM positive group had extrarenal symptoms, compared with only two (7.4%) patients in the IgM negative group. The most frequent symptom was cognitive impairment (3 patients), two patients had short stature, other symptoms (facial dysmorphism, microcephaly, peripheral neuropathy, autism) occurred only once. Two patients had three extrarenal symptoms. One of whom presented at 13 years of age with mental retardation, microcephaly and facial dysmorphism, and he is currently on renal replacement therapy. The other patient presented at 13 years of age with mental retardation, short stature and peripheral neuropathy and she died a year after disease presentation due to severe sepsis.

Three (16.7%) and eight (29.6%) patients were treated with steroids only in the IgM positive and negative group, respectively (*p* = 0.482). In the IgM positive group, patients required more immunosuppressive medications to achieve remission: fifteen patients were treated with Cyclosporine, four with Mycophenolate mofetil, three patients received Rituximab, but the differences were not statistically significant.

### Follow up biopsies

Eighteen follow-up biopsies were performed for thirteen patients. Second biopsy was performed for 8 patients (44.4%) in IgM positive group and for 5 patients (18.5%) in IgM negative group. Follow-up biopsies were performed at a median of 2.08 years (1.52–5.58) in IgM positive group and a median of 2.32 years (2.02–2.32) in IgM negative group with no statistically significant difference (*p* = 1.0). 3 patients (16.7%) in IgM positive group and 2 patients (7.4%) in IgM negative group had a third kidney biopsy performed during their follow-up period. The third biopsy was performed at a median of 5.45 years (2.18–7.06) in IgM positive group and a median of 4.41 years (4.31–4.51) in IgM negative group with no statistically significant difference (*p* = 1.0). Histological disease progression from MCD to FSGS was accounted for in 2 patients. One patient was IgM positive and initially steroid sensitive, and after three adjuvant immunosuppressive medications, he is currently in remission. The other patient was IgM negative, initially steroid-dependent, and after treatment with one adjuvant immunosuppressive medication, and she currently has active disease but with normal kidney function. All four patients who progressed to end stage renal disease revealed FSGS on their first biopsy.

### Outcomes

Duration of follow-up was slightly longer (not statistically significant) in the IgM positive group (median 4.52 years) compared to the IgM negative group (median 3.55 years). Although more patients in IgM positive group had impaired kidney function at last follow-up (22.2% vs 7.4%), the difference did not reach statistical significance.

No statistically significant differences were found in renal outcome between the groups (Table 2). There were four patients with end-stage renal disease: three patients in IgM positive group and one patient in IgM negative group. The median of time of progression to end stage renal disease was 5.53 years, range 0.94–12.37 years. One patient in the IgM negative group is cured and 1 patient in IgM positive group died because of severe sepsis at the age of 14 years after nearly one year of the disease.

**Table 2:** Outcome results

	IgM+ (*n* = 18)	IgM- (*n* = 27)	*p* value
Duration of follow-up, years^c^	4.52 (0.85–13.14)	3.55 (0.17–12.67)	0.194
Age at last follow-up, years^c^	12.86 (2.35–26.22)	9.67 (1.87–24.06)	0.438
Clinical data at follow-up, n (%)			
Hematuria	6/18 (33.3)	9/27 (33.3)	1
Hypertension	13/18 (72.2)	14/27 (51.9)	0.222
Decreased GFR	4/18 (22.2)	2/27 (7.4)	0.199
Outcome, n (%)			
Remission	11/18 (61.1)	13/27 (48.1)	0.543
Active disease, normal kidney function	3/18 (16.7)	12/27 (44.4)	0.063
Active disease, impaired kidney function	2/18 (11.1)	1/27 (3.7)	0.555
Renal replacement therapy	2/18 (11.1)	1/27 (3.7)	0.555

^c^Values are given as median, with the range in parenthesis

## Discussion

IgM deposits have been implied to have diverse significance of the clinical course and outcomes for NS. Some researchers have questioned the significance of IgM deposits alone. It has been proposed, that these deposits could occur due to passive entrapment in the glomeruli due to glomerular sclerosis [9]. This could explain the appearance of IgM deposits in various diseases and conditions. However, it does not explain why similar molecular weight proteins are not detected in co-deposition with IgM [17, 18]. Some studies have shown that IgM deposits bind to specific epitopes in glomeruli and cause complement activation through the classical pathway thus exacerbating glomerular damage [17, 18].

In our study, IgM deposits were found in 40% patients. This result is is higher than in previous studies, but this could be explained by different inclusion criteria. The reported range of IgM deposits in idiopathic NS varies from 24.3% [19] to 62.5% [20], although this last study also included adult patients.

Numerous studies have suggested that IgM deposits in the glomeruli found in patients with NS are associated with poor steroid response. Our data showed higher frequency of steroid resistance in the IgM-positive group compared to IgM-negative (33.3% vs. 18.5%), but the difference was not significant. These results correspond to several other studies [21, 22]. Kanemoto et al. [23] also reported a significantly higher rate of steroid resistance in the IgM positive group as compared with the IgM-negative group (*p* = 0.033). However, this could be due to a larger patient group (IgM+ 30 patients) and the authors do not demonstrate the rate of dense deposits present on electron microscopy.

IgM deposits have been associated with the development of impaired kidney function. Although our data did not produce statistically significant findings, there is a tendency towards impaired kidney function at the last visit in IgM-positive patients. Furthermore, three patients out of 45 (6.6%) progressed to chronic kidney disease (CKD), however the progression of renal disease did not correlate with the presence of the electron dense deposits: none of the 3 patients with electron dense deposits developed CKD. Similar results were found in the Swartz et al. [21] study: of the 26% patients with MCD only one developed CKD. In our study, all 3 patients who are currently on renal replacement therapy had a primary histological diagnosis of FSGS, one of whom was IgM positive. The rate of disease progression to renal failure reported by Mubarak et al. [24] was significantly higher in the IgM-positive group (15.7%) compared with MCD (2.5%, *p* < 0.05), while none of the 7 IgM-positive patients progressed to ESRD as reported by Spreitzer et al. [22]. Nevertheless, these differences could be explained by different inclusion criteria and different indications for the renal biopsy.

We have also taken into account that biopsy results in our IgM-positive patient group were heterogeneous, consisting of MCD, MH and FSGS. In the IgM positive group FSGS was a more common finding than MCD (50.0% vs. 18.5%, *p* = 0.049), therefore a tendency towards poor steroid response and disease progression could be caused by FSGS alone. It has been shown by a few studies that IgM deposits could be also found in patients in FSGS, with a frequency ranging from 19.4% [25] to 90% [26]. In our study, we performed 18 follow-up biopsies and found, that only 1 patient with IgM positive MCD progressed to FSGS, which could be due to the smaller patient group and shorter follow-up period. A similar progression rate was reported by Spreitzer et al. [22]. Our data adds to previously published findings of association of IgM and subsequent finding of FSGS only and do not imply causation.

This study has several limitations. Firstly, this was a retrospective study with a relatively small patient cohort and rather short follow-up period. Secondly, the diagnosis of arterial hypertension is questionable (confusing) since some patients received angiotenzin-converting-enzyme (ACE) inhibitors for the renal protective effect. Therefore, based on the definition of arterial hypertension, all of these patients were included into arterial hypertension group.

## Conclusions

This study has demonstrated that clinical course and disease outcomes did not differ significantly between IgM-positive and IgM-negative groups.

## Abbreviations

ACE: Angiotenzin converting enzyme
CKD: Chronic kidney disease
FSGS: Focal segmental glomerular sclerosis
GFR: Glomerular filtration rate
IF: Immunofluorescence
IgM: Immunoglobulin M
KDIGO: Kidney Disease, Improving Global Outcomes
MCD: Minimal change disease
MH: Mesangial hypercellularity
MN: Membranous nephropathy
NS: Nephrotic syndrome
